# Spatial analysis of BSE cases in the Netherlands

**DOI:** 10.1186/1746-6148-4-21

**Published:** 2008-06-17

**Authors:** Lourens Heres, Dick J Brus, Thomas J Hagenaars

**Affiliations:** 1 Department of Bacteriology and TSEs, Central Veterinary Institute of Wageningen UR, Lelystad, The Netherlands; 2 Alterra, Wageningen University and Research Centre, Wageningen, The Netherlands; 3 Department of Virology, Central Veterinary Institute of Wageningen UR, Lelystad, The Netherlands; 4 Current Address: VION Fresh Meat West, Boxtel, The Netherlands

## Abstract

**Background:**

In many of the European countries affected by Bovine Spongiform Encephalopathy (BSE), case clustering patterns have been observed. Most of these patterns have been interpreted in terms of heterogeneities in exposure of cattle to the BSE agent. Here we investigate whether spatial clustering is present in the Dutch BSE case data.

**Results:**

We have found three spatial case clusters in the Dutch BSE epidemic. The clusters are geographically distinct and each cluster appears in a different birth cohort. When testing all birth cohorts together, only one significant cluster was detected. The fact that we found stronger spatial clustering when using a cohort-based analysis, is consistent with the evidence that most BSE infections occur in animals less than 12 or 18 months old.

**Conclusion:**

Significant spatial case clustering is present in the Dutch BSE epidemic. The spatial clusters of BSE cases are most likely due to time-dependent heterogeneities in exposure related to feed production.

## Background

### BSE case clustering

Disease clustering patterns may provide important clues to the nature of disease transmission. In the context of BSE in cattle, clustering can be defined as cases not being completely randomly distributed amongst farms. We speak of *spatial *clustering if cases are more likely to occur in geographic proximity to other cases. after correcting for spatial differences in density of cattle.

BSE cases first occurred in the Netherlands in 1997. After a peak of 24 cases in 2002 the epidemic has now much declined. In Table 1 we list the chronology of introduction of the different BSE control measures in the Netherlands. The majority of Dutch BSE cases were born after the introduction of a ban on ruminant Meat-and-Bone Meal (MBM) in ruminant feed in 1989, and the results of a case-control study [1] indicate that cross-contamination of ruminant feed with MBM from pig or poultry feed has been the main cause of these cases.

**Table 1:** BSE control measures in the Netherlands.

Year	Measure
1989	Ban on ruminant MBM in ruminant feed
1990	Passive surveillance for BSE; Ban on export of ruminant MBM from GB
1994	Ban on mammalian MBM in ruminant feed
1996	EU ban on import of MBM from GB
1997	Removal and rendering of SRM
April 1999	Zero tolerance for MBM in ruminant feed, i.e. separation of feed production facilities.
December 2000	EU ban on use of MBM and other animal protein in feed of farm animals, excluding dicalcium phosphate, fishmeal, and gelatine for non-ruminants.
January 2001	Active surveillance in cattle over 24 months of age (fallen stock and emergency slaughter) or over 30 months of age (healthy slaughter)

### Possible clustering mechanisms

As the transmission of BSE is at least predominantly feed-borne, case clustering patterns differ from patterns caused by infectious spread via direct transmission between hosts. In particular, none of the farms with BSE in The Netherlands (82 farms in total) has had more than one animal diagnosed [1].

Although this paper deals with a spatial analysis of the BSE case data, we find it useful to discuss the possible clustering mechanisms from the broader perspective of case clustering in general (i.e. not necessarily spatial case clustering). The possible mechanisms generating clustering of BSE cases are numerous. All of them are associated with some form of heterogeneity. For *spatial *clustering of BSE cases, the perhaps most direct mechanism would be regional differences in disease surveillance intensity. However, this mechanism can not apply to the time period we are studying here (from 2001 onwards), during which all cattle over 30 months of age were tested at slaughter, as well as all animals over 24 months of age sent for emergency slaughter or for rendering. It is instructive to distinguish between two remaining possible types of heterogeneity, namely exposure heterogeneity and population heterogeneity.

Exposure heterogeneity, i.e. variation in exposure to infectious feed, may be caused by several mechanisms. The first one is the presence of between-producer differences in the feed processing methods and/or in the origin of MBM. Differences in feed processing may, for example, result in different levels of cross-contamination (after introduction of a ban on MBM in ruminant feed). Differences in origin of MBM can arise if feed producers have different MBM suppliers. Suppliers of MBM may differ in the offal they have processed, and in their processing procedures. The second underlying mechanism is between-farm variation in feeding practice, for example in the amount of feed used or the age of animals at which protein-enriched feedstuff is fed. These first two mechanisms can be viewed as farm-level risk factors, as they relate to the farmer's choice of feed supplier and feeding practice. Regional variation in the presence of these risk factors results in spatial clustering.

The third mechanism causing exposure heterogeneity is aggregation of infectivity within MBM [2]. This mechanism is a more complex heterogeneity between feed "units", where the scale of a unit might range from a bite to bag or batch [2]. Spatial locality in the production and distribution of MBM and feed (i.e. "regional recycling") is a fourth exposure-based mechanism generating clustering. This type of clustering is necessarily spatial. This mechanism is most powerful if the transmission potential is above threshold level [3], such that the regional recycling of BSE infection can produce regional epidemics of BSE infection.

The third type of heterogeneity capable of generating case clustering of a feed-borne disease is population heterogeneity. Genetically determined variations in cattle susceptibility, as well as between-farm variation in age-specific animal replacement probabilities are examples of population heterogeneity that may cause spatial clustering of BSE cases. The latter mechanism may cause clustering because the later an animal is replaced by the farmer, the less likely a BSE infection will remain undetected. We note that spatial variation in population density can not (trivially) lead to spatial clusters of BSE cases in our analyses, as the testing methods applied in this paper account for differences in population density.

By theoretically working out the characteristics of the clustering patterns associated with the different possible mechanisms, expected differences in these patterns can be identified. Subsequently, if the data are informative enough, then these differences may allow us to exclude certain candidate clustering mechanisms, thus producing insight into the properties of the transmission cycle. For example, Hagenaars et al. [2] used mathematical modelling of feed-borne transmission to show that infectivity aggregation within MBM would give clustering characteristics different from those observed in the BSE epidemic in Great Britain (GB). Thus infectivity aggregation in feed could be excluded as underlying mechanism of BSE clustering in GB. Feed processing heterogeneities and heterogeneity in feeding practice (i.e. variation in the per-animal feed uptake) remained candidate mechanisms in GB, as they do produce clustering patterns consistent with observed patterns [2].

In this paper we will focus on the question whether there is spatial clustering present in the Dutch BSE epidemic. Scientifically explaining the results in terms of underlying mechanisms was impossible due to insufficient data. However, in the discussion we will argue that the patterns observed are likely to be generated by exposure heterogeneities related to feed production that occurred during a limited period.

### Observed BSE clustering patterns

In order to be able to relate our results for the Dutch epidemic to the ones in GB, France, Switzerland, Ireland, and Spain, we now briefly review the BSE clustering patterns observed in those countries. For a comparison with The Netherlands, the most interesting clustering patterns are those in animals born after a ban on ruminant MBM in ruminant feed was introduced in the country.

Both in GB and in France, farm type (dairy versus beef suckler) has been identified as an important risk factor [4,5]. Furthermore, holding size was identified as a risk factor in GB [4,6] and Switzerland [7]. In several countries including Spain, regional differences or spatial clusters were found in BSE incidence [4,8-18]. These spatial clustering patterns were hypothesized to be generated by various kinds of exposure heterogeneity. The spatial clustering pattern of BSE in GB has been studied in [19-21]. Donnelly and Ferguson [21] found that clustering is present at all spatial scales ranging from 100 to 819 km. They concluded that there must have been a number of processes, operating at different scales, causing exposure heterogeneity. The spatial clustering pattern of BSE in Switzerland has been studied in two papers [14,16]. Swiss spatial clusters of cases born after a ban on MBM in ruminant feed were found to be correlated with higher pig densities, consistent with the assumption that these cases were due to cross-contamination of cattle feed with feed containing meat and bone meal, intended for other species such as pigs [16]. Sheridan et al. [17] detected spatial BSE clusters in Ireland, and provided support for the hypothesis that these are associated with feed-producer related heterogeneity. Finally, spatial clustering of BSE was observed in France [12,13,15,22,23] and some evidence was presented for correlations between the BSE clustering pattern and the density of pigs and/or poultry, consistent with the hypothesis that cross-contamination caused recycling of the BSE infection in French cattle born after a ban on MBM in ruminant feed [22].

### Research question

In Great Britain evidence was found that most BSE infections occur in animals less than 12 or 18 months old [24,25]. This implies that the BSE cases born in a given period of 1 to 1.5 year provide information of the BSE exposure levels during that period. It also implies that any spatial case clustering arising from exposure heterogeneities lasting for about a year only, would be best detectable by analyzing the case data on a cohort-by-cohort basis. Such an analysis gives insight into where and when exposure levels were relatively high. Finally, a cohort-specific analysis is motivated by the possibility that when several cohorts are analyzed together, spatial clusters can be obscured if the elevated exposure is not persistent but shifts across the study area. Research questions of this study therefore are:

1. Are the BSE cases in the Netherlands spatially clustered, and if so where are these clusters located?

2. Are BSE cases born in a given period of one year spatially clustered, and if so where are these clusters located?

3. Can we relate any observed case clustering to one or more possible underlying heterogeneities?

## Methods

### Case and population data

We analyzed the BSE cases in the period from the start of active surveillance (1 January 2001) until 31 December 2004 (69 cases in total). The locations and sizes (number of animals) of all Dutch dairy farms were available from the agricultural census data [26]. The case data for 2005 and 2006, five cases in total, were not included as no census data were available yet for these years. Table 2 lists how the 69 cases are distributed over birth cohorts, and over age at detection. The estimated local population sizes were obtained on the basis of the average number of cows older than two years in the period 2001–2004, and an estimated age distribution (used for the birth-cohort specific analysis). This age distribution, shown in Table 3, is based on the population structure observed on 100 Dutch dairy farms, randomly selected from the full Dutch dairy farm population. As the average age of dairy cattle in Dutch herds has been stable during the last 5 years and no major changes occurred in the dairy industry during these years, we assume the age distribution to be the same for the different birth cohorts. The average and median age observed in the random set of farms were very similar to those for another set of farms, comprising the Dutch BSE herds and more than 120 control herds [1]. This provides confidence that the estimated age distribution is a good representation of the Dutch dairy population. We assume that there are no regional differences in animal replacement policies, as the dairy farm management culture is known to be highly uniform across the Netherlands.

**Table 2:** Dutch BSE cases. BSE cases in the period 2001–2004, by cohort and by age at detection.

**Cohort**	88	91	92	93	94	95	96	97	98	99
**Number of cases**	1	2	2	2	5	7	32	12	5	1
**Age at detection**	4	5	6	7	8	9	10	11	12	13
**Number of cases**	9	21	21	9	3	2	1	0	2	1

**Table 3:** Cattle age distribution. Age distribution of cattle, based on population data from 100 randomly selected Dutch dairy farms.

**Age**	**Total dairy population**	**Dairy > 2 yr**
0-1	20.8%	
1-2	18.7%	
2–3	16.2%	26.83%
3–4	13.3%	21.92%
4–5	10.1%	16.62%
5–6	8.1%	13.38%
6–7	5.3%	8.76%
7–8	3.7%	6.09%
8–9	2.0%	3.26%
9–10	1.0%	1.67%
10–11	0.4%	0.73%
11–12	0.2%	0.37%

Table 4 shows summary statistics of the data. The majority of BSE cases were born, raised and culled at the same farm. In the few instances where this was not the case, we used the location of the farm where the animal stayed during the first half year of its life.

**Table 4:** Population size and case incidence. Mean total population size and overall case incidence for the study period 01/01/2001 – 31/12/2004.

Size of the population older than 2 yr (2001–2004 average)	1815449
Total number of cases in the study period	69
Average yearly number of cases per 100000 cattle older than 2 yr	0.95

### Testing for clustering

We first tested whether clustering is present (global clustering test), and then detected and tested local clusters. To test whether spatial clusters are present, we used Diggle and Chetwynd's method [27]. The Kulldorff scan test [28,29] was used to detect and test local clusters. Both methods account for spatial variation in population density, which may cause deviations from a completely random spatial point pattern. Both the global and local clustering tests were performed in two ways: on the overall case data and on the case data for each birth cohort separately. In order to see if and how the results of the cohort-wise analyses are influenced by the definition of the cohorts, we have carried out two variants of these analyses: one using calendar years as birth years and one using 1 July to 30 June periods as birth years. Hereafter, birth cohorts starting on 1 January will be denoted by a single year (e.g. 1996), while birth cohorts starting on 1 July will be denoted by two years (e.g. 1996/1997).

The K-functions in Diggle and Chetwynd's method were estimated for distances 5 km, 10 km, ..., 50 km. For the K-function of the controls we selected 2000 controls from the population at risk by random sampling with probabilities proportional to size. We used the R-package SPLANCS for this global clustering test [30]. 999 Monte Carlo runs were performed to test the null hypothesis of no spatial clustering.

To reduce computing time of the Kulldorff scan test, the number of cases and animals at risk were aggregated to totals for 1 km^2 ^square grid cells. For 999 Monte Carlo runs computing time was 7 h. 49 min when the original farm-level data were used, whereas with aggregated data this was 1 h 24 min (using a standard computer with a 2.60 Ghz processor and a RAM of 504 MB). This enabled the increase of the number of Monte Carlo runs to 9999 (computing time 10 h 23 min). The effect of the spatial aggregation on the location, size, and *p*-value of the clusters was negligible. The systematic scanning of the spatial data and the statistical testing were carried out using the freeware SaTScan, version 7.0.3 [29]. Data were treated as spatial data (no time dimension). The population size changes over the years, and therefore the time-averaged population size was used in the analysis. The centres of the 1 × 1 km grid were used as centres for the circular windows (19030 nodes). The maximum circle size was set by requiring the window to contain at most 25% of the population. Note that the shape of the scanned neighbourhoods is often not circular but irregular, especially near the nation's boundary. However, the shape of the neighbourhood is immaterial; what counts are the sizes of the populations at risk inside and outside the scanned neighbourhood. The *p*-values were computed from 9999 Monte Carlo replications.

## Results

For all distances considered, the difference between the estimated K-functions of cases and controls based on all 69 cases is far beyond the upper confidence bound, calculated as 1.96 times the standard error of the difference. This indicates strong spatial clustering at all distances, which is confirmed by the very small *p*-value of the test statistic (*p *= 0.001, Table 5). Also for the 1996 birth cohort (32 cases) and the 1996/1997 birth cohort (25 cases) the differences between the K-functions of cases and controls are well above the upper confidence bounds, indicating strong spatial clustering of BSE cases for these birth cohorts. The *p*-values of the test statistic are again 0.001 (Table 5). For the 1995/1996 birth cohort (17 cases) the difference of the K-function is in between the confidence bounds for all distances considered, indicating that there is no significant spatial clustering for this cohort, which is confirmed by the large *p*-value of 0.360. For the remaining birth cohorts the numbers of cases are considered too small to allow valid testing of global clustering.

**Table 5:** Results of a global clustering analysis using Diggle and Chetwynd's test. The *p*-value is of Diggle and Chetwynd's test statistic *D*_*c*,*k*_.

	**All cohorts**	**1995/1996**	**1996**	**1996/1997**	**1997**
*p*-value	0.001	0.361	0.001	0.001	0.001

**Figure 1:**
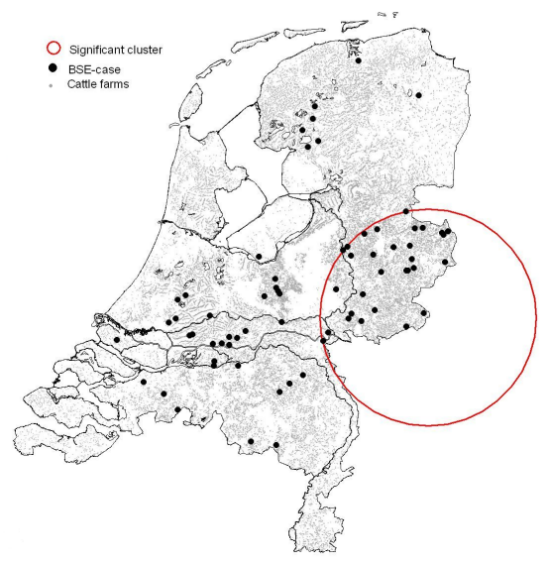
**Spatial BSE case clusters**. Spatial clusters of BSE cases detected in an analysis not accounting for birth cohort.

**Figure 2:**
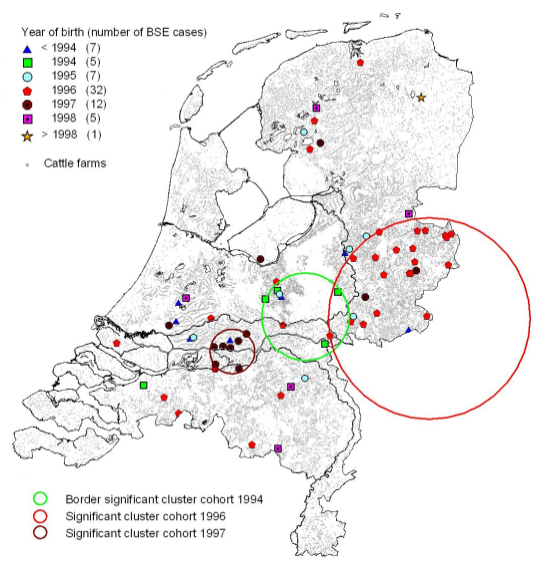
**Cohort-specific spatial BSE case clusters**. Spatial clusters of BSE cases detected in a cohort-wise analysis in the 1 January 1994 – 31 December 1994 birth cohort (red), the 1 January 1996 – 31 December 1996 birth cohort (blue) and the 1 January 1997 – 31 December 1997 birth cohort (magenta).

Figures 1 and 2 and Tables 6 and 7 show the results of the local clustering tests (Kulldorff scan tests). The analysis not accounting for birth cohort (Figure 1, Table 6) resulted in one significant cluster (*p *= 0.0387). A second cluster was found that was not significant (*p *= 0.1795). Cohort-wise clustering analysis (Figure 2, Table 7) resulted in significant clusters for the birth cohorts 1994, 1994/1995, 1996, 1996/1997, 1997. and 1997/1998 (Table 7). The 1996 cohort cluster in the eastern part of the Netherlands (Figure 2) coincides with the first cluster of the combined analysis (Figure 1). The clusters of the 1996 birth cohort and of the 1996/1997 birth cohort overlap to a large extent: they share 15 BSE cases (which are all born in the period 1 July 1996 – 31 December 1996). All 6 cases of the 1997/1998 cohort cluster are born between 1 July 1997 and 31 December 1997 and are also part of the 1997 birth-cohort cluster. This cluster is located in the middle-west part of The Netherlands. It corresponds to the second (non-significant) cluster in the combined analysis. The 1994 and 1994/1995 cohort clusters have two BSE cases in common, and are situated in the centre of the Netherlands.

**Table 6:** Results of a local clustering analysis using Kulldorff's scan test. All 69 BSE cases (no distinction between cohorts).

	**First cluster**	**Second cluster**
Population size inside cluster	329786	26611
Number of cases in cluster	30	8
Expected number of cases	12.53	1.01
Annual number of cases/100000	2.3	7.5
Observed/expected	2.393	7.910
Relative Risk	3.47	8.82
Log likelihood ratio	11.75	9.93
*p*-value	0.0387	0.1795

**Table 7:** Results of a local clustering analysis using Kulldorff's scan test. Cohort-wise analysis.

**Cohort**	**Population size inside cluster**	**Total number of cases**	**Number of cases inside cluster**	**Log likelihood ratio**	***p*-value**	**Centre of cluster**	**Radius of cluster (km)**
1994	55545	5	4	9.941	0.0484	(181500,444500)	25.079
1994/1995	69535	5	3	13.17	0.0053	(162500,456500)	4.472
1995	No significant clustering						
1995/1996	No significant clustering						
1996	145764	32	20	16.55	0.0007	(252500,443500)	57.870
1996/1997	171601	25	18	18.40	0.0002	(252500,443500)	57.870
1997	206348	12	7	19.60	0.0001	(139500,424500)	12.806
1997/1998	242090	10	6	17.04	0.0004	(139500,424500)	12.806

**Figure 3:**
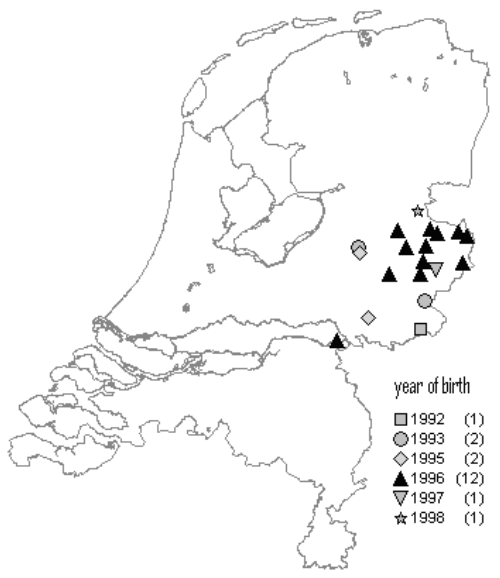
**Case farms supplied by one particular feed producer**. Locations of all farms that had BSE cases and were supplied by feed producer A [1].

**Figure 4:**
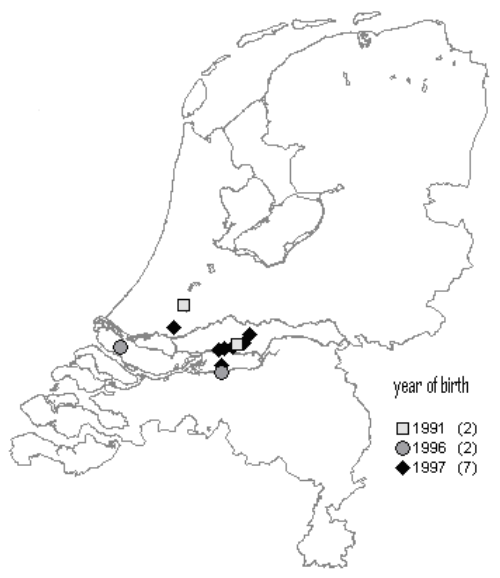
**Case farms supplied by one particular feed producer**. Locations of all farms that had BSE cases and were supplied by feed producer K [1].

In a non-temporal, non-spatial analysis of Dutch case-control data [1], it was found that the "group of feed producers" is a significant risk factor for BSE. This previous study suggested that the feed-production heterogeneities have most likely arisen due to both origin of MBM and cross-contamination on mixed production lines. We were therefore interested in the question whether feed suppliers can be identified that are associated with the three clusters of the cohort-wise analysis. For the farms with BSE cases we have collected feed supplier information. The four farms in the cluster of the 1994 birth cohort were supplied by three different (farmer owned) feed mills. In each of the two cohorts 1996 and 1997, we observed that one particular feed mill is more frequently used as supplier inside the spatial cluster than outside. The cluster of 1996 birth cohort contained 20 cases, 11 of which were supplied by producer A (proportion = 55%). In this birth cohort there were 12 cases outside the cluster, and only one of these was supplied by A (proportion = 8.3%). Using Fisher's exact test on these proportions revealed a significant association between the 1996 cluster and feed producer A (*p *= 0.021, two-tailed probability). Note that the association with the 1996 cluster cannot serve as evidence for producer A being associated with BSE risk. However, producer A did belong to the group of feed producers found to be associated with BSE risk in a former study [1]. The 1997 case cluster contained seven cases, five of which were supplied by producer K (71.4%), another member of the group of feed producers found to be associated with BSE risk in the former study [1]. In the 1997 birth cohort there were five cases outside the cluster, and two of these were supplied by K (40%). These numbers do not reveal a significant association between the 1997 cluster and feed producer K (*p *= 0.558, Fisher's exact test). Figure 3 and 4 show the locations of all farms with BSE cases supplied by the producers A and K, respectively.

## Discussion

We have found three spatial case clusters in the Dutch BSE epidemic. The clusters are geographically distinct and each cluster appears in a different birth cohort. The fact that we found stronger spatial clustering when using a cohort-based analysis, is consistent with the evidence that most BSE infections occur in animals less than 12 or 18 months old [24,25]. As a result of the infection at a young age, temporal changes in BSE exposure are seen most clearly by comparing cohort-wise incidence levels. The fact that each of the three significant clusters in the cohort-based analysis occurs in a different birth cohort, suggests that the causes of the enhanced infection levels each occurred within a limited time frame of at most about a year.

In the Introduction we discussed the possible mechanisms that may produce clustering of BSE cases. The candidate mechanisms that could underlie the observed spatial clusters in the Netherlands are feeding practice and on-farm cross-contamination, and heterogeneities in rendering and feed processing. Local recycling is not likely as the number of rendering plants in the Netherlands was as low as two and each of these supplied nationwide to feed producers. Population heterogeneity is unlikely because in previous work [1] neither genetic differences between regions have been found nor differences in management. Population heterogeneity as a cause of spatial clustering is also unlikely in view of the limited time frame in which the causes of the clusters seem to have been present.

In the same previous work, the factor "group of feed producers" was found to be a significant risk factor in a non-temporal, non-spatial analysis of case-control data [1]. Based on this previous result we therefore interpret the observed clustering to be at least in part due to regional differences in feed production. As the information from the previous study suggested, the feed-production heterogeneities have most likely arisen due to both origin of MBM and production on mixed production lines. Feed producers were different in their sourcing of MBM and the use of mixed or dedicated production lines. Separation of production lines was not obligatory up until 1999 (Table 4), and both producers A and K have used mixed production lines up until then. Variation in feeding practice (i.e. between-farm variation in the per-animal feed uptake) is a less likely mechanism to have contributed to the clustering, as the amount of feed fed was not significantly associated with BSE in the previous study. Furthermore, a contribution due to on-farm cross-contamination as a consequence of mixed farming has not been detected. Indications against such a contribution are the fact that the 1997 cluster is in an area with small numbers of pigs and the observation that the southern part of the Netherlands with dense populations of pigs has a relatively small number of BSE cases.

Also in some other European countries where spatial clusters of BSE have been found, the most likely mechanisms were suggested to be related to exposure heterogeneity [4,10-19,23]. The feature of different spatial clustering occurring in different birth cohorts has also been observed elsewhere in Europe [10,12,14,15]. In Switzerland, France and Great Britain it was difficult to distinguish effects due to feed processing differences from those arising from differences in feeding practices between mixed farms and farms with only ruminants, because typically feed producers with mixed production lines and mixed farms were spatially correlated. In a recent analysis of Swiss data for the period after the introduction of a ban on MBM in cattle feed, Schwermer et al. [31] found evidence of spatial association between BSE cases and feed producers where cattle feed was found MBM positive by cross-contamination. Cross-contamination in the feed-production process was also implicated in a recent study by Paul et al. [23], in which a spatial analysis of the French feed industry and BSE case data showed that BSE risk in France after a ban on MBM in ruminant feed is spatially linked to the use of MBM in non-ruminant feed.

## Conclusion

We have identified three spatial case clusters in the Dutch BSE epidemic. The clusters are geographically distinct and each cluster appears in a different birth cohort. In a former study the factor "group of feed producers" was found to be a significant risk factor in a non-temporal, non-spatial analysis of case-control data [1]. Based on this result we interpret the spatial clustering observed here to be at least in part due to regional differences in feed production. We have found that the 1996 cluster is significantly associated with one particular feed producer. These results suggest that many of the BSE cases in the Netherlands may have been due to incidental infectivity releases, that are both temporally and spatially confined.

## Authors' contributions

LH conceived of the study, collected the feed supplier information of BSE case farms, participated in the clustering analysis and the interpretation of the results, and drafted the manuscript. DJB identified the relevant statistical methods for the clustering analysis, carried out the statistical calculations, participated in the interpretation of the results and helped to draft the manuscript. TJH participated in the clustering analysis and in the interpretation of the results, helped to draft and finalized the manuscript. All authors read and approved the final manuscript.
